# Are you ready? A systematic review of pre-departure resources for global health electives

**DOI:** 10.1186/s12909-019-1586-y

**Published:** 2019-05-22

**Authors:** Anna Kalbarczyk, Emily Nagourney, Nina A. Martin, Victoria Chen, Bhakti Hansoti

**Affiliations:** 1 Johns Hopkins Center for Global Health, Baltimore, USA; 2 Johns Hopkins Bloomberg School of Public Health, Baltimore, USA; 3 Johns Hopkins School of Medicine, Baltimore, USA

**Keywords:** Predeparture preparation, Travel, Global health, International elective, Short-term training

## Abstract

**Background:**

There has been an exponential increase in the offering of short-term international field experiences in recent years in response to student demands for global health opportunities. Pre-departure preparation is an essential component to equip trainees with the adequate safety, wellness, and cultural competence needed to engage in a meaningful and mutually beneficial elective. This review seeks to quantify the plethora of pre-departure preparation training available to public health, clinical, and undergraduate trainees across the continuum of education for short-term experiences in low-and middle-income countries (LMICs).

**Methods:**

We performed a systematic review of Pubmed, Embase, Web of Science, Scopus, and Ovid Global Health in February, 2018. A three-concept search was employed and included “global or international health”; “education or preparation of personnel/students”; and “field programs or travel.” The study teamed used PRISMA reporting guidelines to conduct title and full-text reviews and conduct data extraction and analysis.

**Results:**

The search returned 2506 unique articles. Of these, 55 met inclusion criteria and were included in the final review. Ninety one percent (91%) of articles focused on pre-departure trainings for medical students and residents. Nine thematic domains for short-term international field experiences emerged; culture, safety, and project-specific knowledge were the most frequently covered domains while mentorship, professionalism, and emotional wellness and culture shock were least common. Approximately half (53.3%) of studies specifically evaluated the pre-departure component of the international experience using a survey or evaluation form. Recommendations emerged from these evaluations including early engagement with international partners, inclusion of self-reflection exercises and site-specific content, and utilization of interactive approaches in learning. Some institutions face barriers to conducting pre-departure preparation such as lack of dedicated faculty, finances, and institutional support.

**Conclusions:**

Interest in pre-departure training for international experiences is growing but few programs conduct and publish evaluations of these trainings. Pre-departure trainings should be developed in partnership with receiving institutions and faculty and incorporate critical self- reflection throughout the experience. In addition to the experience itself, institutions need to evaluate these curricula to better understand how they influence trainees’ capacity to effectively engage in LMIC settings.

**Electronic supplementary material:**

The online version of this article (10.1186/s12909-019-1586-y) contains supplementary material, which is available to authorized users.

## Background

Student demand for global health experiences has increased substantially over the past decade and many institutions now have pan-university institutes and centers that provide monitoring and oversight for global health education and training [1, 2]. An increasingly popular option offered by training institutions are global health field experiences, designed for trainees across the education continuum, from undergraduate students to medical residents [2]. These field experiences within academic contexts often take the form of a trainee from a high-resource context travelling to a site in a lower-resource setting. The trainee embeds within a local organization or host institution to complete a set of pre-defined activities for a set amount of time and returns to their home training institution at the end of the field experience. Many institutions encourage such experiences as a way to translate theoretical knowledge into applied practice within a specific context.

As global health field experiences grow in popularity, so too does the visibility of pre-departure preparation programs. In the context of a global health field experience, we define pre-departure preparation broadly as any didactic (e.g. health and safety training) or logistical (e.g. travel health clinic visit) steps taken to ensure trainee safety and competence for the experience. Adequate review of a country’s social, economic, and cultural context, critical debate, and sensitization, whether through pre-departure preparation or longer term educational programs, could prepare trainees to better reflect on possible negative impacts of their elective, which include perpetuating harmful narratives and subsequent consequences of inequity and disenfranchisement for students, institutions and international collaborators [3–6]. Pre-departure preparation also provides an important backdrop to address several criticisms of global experiences, including exploiting poverty, promoting a postcolonial narrative, inequitable exchanges, lack of cultural grounding and critical thinking, and providing limited resources for long-term capacity building of partners and communities [7–13]. One recent survey of host institution partners indicated the importance of trainee understanding of the local context and culture, in addition to technical or project-specific knowledge [14] - material that should be covered in pre-departure preparation courses.

While numerous guidelines and frameworks for engagement in global health training have been articulated [15–19] and several groups have compiled resources for criteria to consider when selecting appropriate overseas experiences [20, 21], there is little published guidance on the best practices for designing pre-departure preparation curricula specific to global health experiences. Several professional groups such as American College of Physicians and Association for Medical Education in Europe have publicly recognized the need for quality pre-departure preparation, but focus on preparation for clinical electives, which may not be translatable to broader global public health training experiences [22–25].

We conducted a comprehensive, systematic review on the current approaches and best practices in pre-departure training for clinical and public health students participating in global health field experiences in low- and middle-income (LMIC) settings. For the purpose of this review, we focus on shorter electives – defined as less than 11 months in duration – since these reflect the most common elective formats in medical, undergraduate, nursing, and public health educational training [26–28].

## Methods

The study team conducted a systematic review of the literature in Pubmed, Embase, Web of Science, Scopus, and Ovid Global Health and employed a thematic analysis of the data. A comprehensive search strategy was developed using a combination of Medical Subject Headings (MeSH) terms and focused text. A three-concept strategy was developed that included, “global or international health,” “education or preparation of personnel/students,” and “field programs or travel.” Search terms are included in Additional file 1.

A systematic approach adherent to the Preferred Reporting Items for Systematic Reviews and Meta-Analyses (PRISMA) guidelines was used to conduct this review [29]. After the removal of duplicates, each study title and abstract were screened to identify articles that were related to pre-departure training or preparation in LMICs. Articles were excluded if they were not written in English, non-healthcare focused, included experiences in high-income countries, focused on non-global health electives, or a format that provided insufficient depth of information (i.e. conference abstracts). Covidence© software (Veritas Health Innovation, Melbourne, Australia), an online platform for data screening and extraction, was used to conduct the primary stages of the review [30].

The remaining articles were further screened by two independent reviewers using the full text Articles were included if they provided specific information on preparatory resources for global health students traveling to LMICs and were available in the English language. A third reviewer garnered consensus and resolved conflicts during both the screening process.

Two independent reviewers then conducted data extraction on the final set of included articles. Data was extracted in Google Forms© and exported to Microsoft Excel© (Microsoft Corporation, Washington, USA) [31]. A third reviewer provided consensus where there were discrepancies in the extracted data. During the extraction process reviewers included a brief description of the article, data source, commonly cited competencies, trainee institution type, trainee population, whether or not the article provided information on a pre-departure course or training element, barriers to predeparture preparation, modes of preparation, and any reported outcomes. Articles that reported outcomes were further reviewed to identify recommendations and best-practices. Each paper was then reviewed by the authors to determine the preparatory thematic domains reported in the articles and to discuss heterogeneity in resources available and potential gaps in the pre-departure preparation process. During a team meeting, consensus was built around the types of thematic domains discussed in the articles and a strategy was developed on how to map these domains to each article. For example, a study could be categorized as using the “safety” domain only if it mentioned that safety was part of the preparatory material provided to trainees.

### Data analysis

Upon completion of data extraction, pre-departure training programs were further grouped into predefined global health thematic domains. The domains were derived using consensus between authors and informed by themes mentioned during in-depth review of the individual studies. Specifically, we identified nine thematic domains which are further defined in Table 1: Culture, Emotional Wellness and Culture Shock, Ethics, Language, Mentorship, Personal Health, Professionalism, Project-specific Knowledge, and Safety. For each study included in the review, two authors reviewed each article and applied the relevant thematic domains for comparison in Microsoft Excel© (Microsoft Corporation, Washington, USA). Any conflicts in assigning domains were reviewed by the study team.

**Table 1:** Travel preparation competencies and their definitions

Competency	Definition
Culture	cultural competency training
Safety	crime prevention, emergency or evacuation protocol and first aid
Project-specific knowledge	procedural skills (clinical or non-clinical), detailing scope of work, and logistical support
Ethics	certified scope of work, responsibility to local collaborators and community members, and power dynamics
Travel medicine	recommended immunizations, post-exposure prophylaxis, and health insurance coverage abroad
Language	formal or informal language courses and fulfilling language competency requirements
Mentorship	expectations, feedback delivery, and communication plan
Professionalism	clinical communication skills, expectations of the patient-provider interaction
Emotional wellness and culture shock	mental health, mental processing of experiences, homesickness

This is a systematic review with no human subject participants and thus ethical approval for this research was not sought.

## Results

The search was conducted in all aforementioned databases on February 28, 2018. This yielded a total of 4625 results and once duplicates were removed 2447 studies were available for title screening. Of these, 2062 were excluded during the title screening phase.

**Fig. 1:**
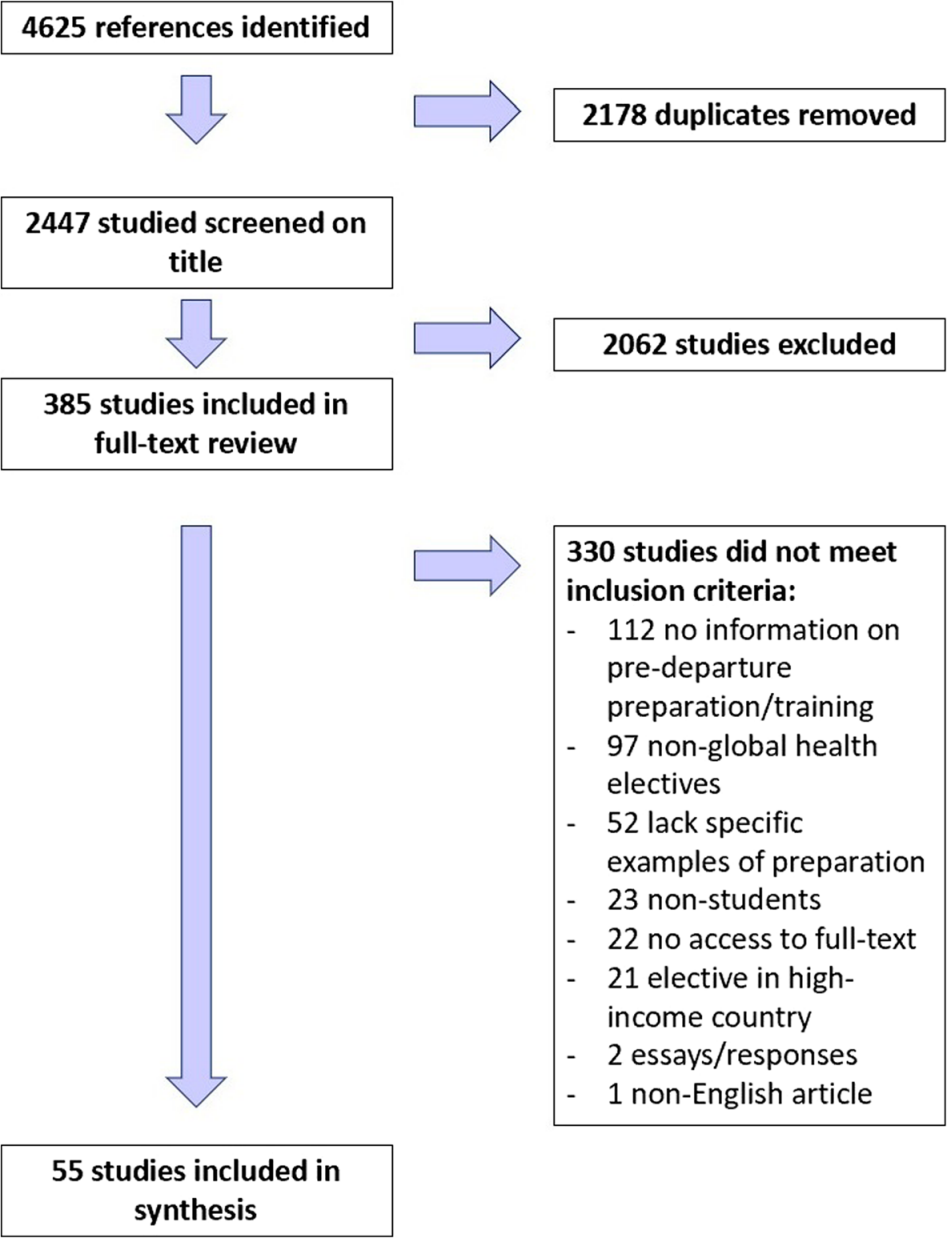
PRISMA diagram

Three hundred and eighty-five full-texts were further reviewed. Of these, 330 were excluded for the following reasons: no information on pre-departure preparation (*n* = 112), non-global health electives (*n* = 97), lack of specific examples of preparation (*n* = 52), non-student related (*n* = 23), no access to full-text (*n* = 22), electives in high-income countries (*n* = 21), and non-English (*n* = 1). A total of 55 studies were included in the final review. A PRISMA diagram (Fig. 1) provides a representation of the overall results for the systematic review process. Additional file 2 shows the studies included in this review and displays key findings.

Of the 55 articles included, 21 were single program evaluations [32–52], 17 were multiple program evaluations [4, 53–68], nine were commentaries or guidelines [69–77], and eight were reviews [26, 78–84]. Articles included in the systematic review were published between 1996 to 2018, with 33 articles (60%) published within the last 5 years.

Ninety-six percent (*n* = 53) of the trainee institutions represented in the articles were universities and hospitals, while two were non-governmental organizations: Health Education and Relief Through Teaching (HEARTT) and the Council of Emergency Medicine Residency Directors (CORD) [67, 83]. HEARTT facilitates the placement of emergency medicine and pediatric residents from US teaching hospitals into clinical rotations at the John F. Kennedy Medical Center in Liberia [67]. CORD, a US-based organization, holds an annual Academic Assembly during which select discussions have focused on evaluating how global health experiences are integrated into emergency medicine residency training programs [83].

The majority of (*n* = 33) articles discussed global health elective preparation for medical students [33–35, 37, 39–47, 49, 50, 52–54, 57, 59, 60, 62, 63, 65, 66, 71, 72, 76, 79–82, 84], followed by preparation for residents and fellows (*n* = 17) [4, 26, 33, 35, 49, 55, 56, 64, 65, 67–69, 71, 73, 75, 79, 81, 83], nursing students (*n* = 6) [36, 44, 54, 58, 66, 77], undergraduate students (*n* = 6) [38, 44, 54, 58, 70, 74], and other graduate students, which included midwifery, pharmacy, physiotherapy and public health students (*n* = 8) [32, 35, 44, 48, 51, 61, 66, 78]. Twelve papers included information about multiple trainee populations [32, 33, 35, 44, 49, 54, 58, 65, 66, 71, 79, 81].

### Thematic domains

Nine thematic domains for global health elective preparation emerged from this systematic review. Each domain and its definition can be seen in Table 1. The most commonly addressed themes were Culture (*n* = 37) [4, 26, 32, 34–36, 38, 40–46, 48, 51–53, 55–57, 63–66, 68–74, 76–81, 83], Project-specific Knowledge (*n* = 30) [32, 34, 36, 40–46, 48, 50, 52, 54, 57–59, 61, 62, 64, 65, 67, 69–71, 74, 76–79] and Safety (*n* = 29) [4, 34–38, 41–44, 53, 54, 57, 59, 61, 63, 65, 66, 68–70, 74, 76–78, 80–83]. Far fewer articles discussed Mentorship (*n* = 11) [26, 44, 59, 69, 71, 74, 76, 77, 79, 81, 83], Professionalism (*n* = 8) [4, 26, 44, 59, 68, 76, 81, 83] and Emotional Wellness and Culture Shock (*n* = 7) [4, 36, 38, 44, 54, 55, 63]. Eighty-four percent of articles (*n* = 46) addressed multiple thematic domains. Nine articles (16.4%) included six or more domains [4, 44, 59, 65, 68, 71, 74, 76, 77], while only one study included eight domains [76]. No studies included all nine domains and 11 articles (20%) included only one domain [33, 39, 47, 49–51, 55, 60, 67, 75, 84].

A display of the thematic areas addressed in the articles for different types of trainees is shown in Table 2. Of those articles discussing pre-departure training for medical students, more than 50% covered culture, project-specific knowledge, safety, personal health, and ethics. Articles assessing pre-departure training for other types of graduate students also focused on these topics though fewer (only 33.3% of articles) mentioned ethics. None of the articles assessing pre-departure for nursing students discussed ethics. Articles on pre-departure preparation for residents and fellows focused primarily on culture (70.6%). All other thematic domains were discussed in less than half of the articles for this trainee population. Articles on undergraduate preparation tended to more frequently cover more of the thematic domains; six of the nine domains were addressed in 50% or more of these articles. Undergraduates and nursing students were the only trainee groups for which more than half of the articles discussed emotional wellness and culture shock; for other trainee groups this domain was included in only 9–11% of articles.

**Table 2:**
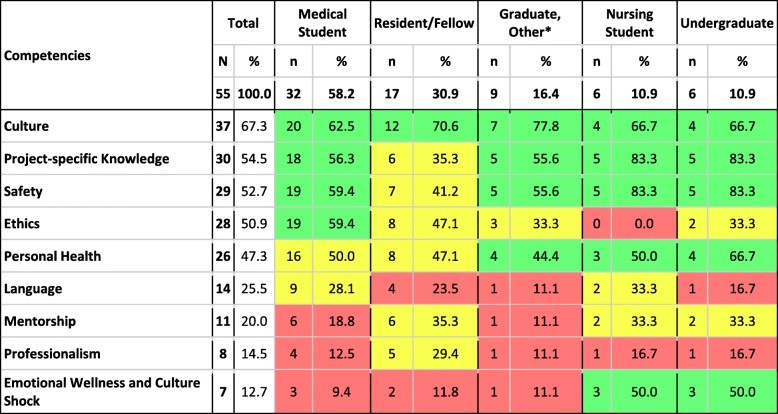
Nine preparation competencies and their frequency by trainee population

*Other graduates included 3 Pharmacy, 2 Physiotherapy, 2 Public Health, 1 Dental, and 1 Midwifery

**12 papers included information about multiple trainee types

### Evaluation

Thirty studies conducted a formal evaluation or assessment of the short-term experience as a whole [32–39, 41–44, 46, 48, 50–53, 55–64, 67, 68]. Of those 30, 16 (53.3%) studies specifically evaluated the pre-departure component using a survey or evaluation form [36, 38, 48, 50–52, 55, 58–64, 67, 68]. Trainees reported that the pre-departure component improved knowledge (*n* = 9) [36, 38, 51, 52, 55, 58, 59, 64, 67], was helpful for their trip preparation (*n* = 8) [48, 50–52, 55, 59, 64, 67], equipped them with clinical or practical skills (*n* = 4) [48, 50, 62, 64], and increased their confidence (*n* = 3) [48, 63, 67]. Two studies demonstrated that predeparture training, specific to exposure to bodily fluids and implementation of post-exposure prophylaxis (PEP) protocols, reduced exposure among trainees [33, 50]. Another study discussed the importance of post-exposure resources and indicated that it is currently an important gap in global health clinical elective infrastructure [68].

All studies, regardless of trainee population, recommended formal pre-departure training as a conclusion of the evaluation. Almost all papers that presented evaluation results (29/30) provided specific recommendations for pre-departure training in their discussion. These recommendations advocated for mandatory, long-term, comprehensive, and interactive pre-departure training curricula and included suggestions to incorporate the relevant health knowledge necessary to travel abroad and/or support patients while abroad with training on skills, attitudes and cultural awareness. Specific recommendations for improved pre-departure training included:

- Conducting critical reflection exercises, both prior to and after the short-term experience [38, 49, 52]. This should include a formal debriefing process after students return from their experience [32, 35, 39, 63].
- Use of interactive and/or case-based approaches [37, 43, 48, 57, 58, 64]. Simulation was also shown to improve attitudes and skills among trainees, not just medical knowledge [64].
- Working with an invested supervisor or mentor both off [52] and on-site [39] and having faculty dedicated to pre-departure preparation [84]
- Building in cultural and health material that is not only country-specific, but also site specific (i.e. cultural competency to the location) [45, 51]. Two articles further argued for the importance of building in some level of language requirement, depending on the location of the experience and setting in which the trainees will work [35, 36].
- Engaging more deeply with collaborators and hosting institutions throughout the process, including during the development of the elective and pre-departure training components [32, 37, 52, 60, 70]. Specifically, these articles discussed using partnership models when sending students overseas and ensuring equitable engagement with both the receiving institution and receiving communities.

### Delivery platform

Studies not only focused on the utility of pre-departure training, but also the platform for training delivery. Articles discussed a variety of preparation modalities including in-person training (*n* = 16) [26, 34, 36, 38, 40–43, 48, 52, 57, 59, 63, 66, 77, 81], didactic training (*n* = 11) [32, 45, 46, 50, 51, 53, 56, 66, 67, 69, 79], text-based training (*n* = 10) [4, 33, 40–42, 54, 71–73, 76], simulation training (*n* = 4) [50, 55, 64, 67], and web-based training (*n* = 3) [53, 56, 58]. Six articles did not specify the delivery modality employed by the study for pre-departure preparation [35, 44, 49, 68, 70, 80], while three articles explicitly mentioned that preparation is dependent on multiple factors, including the elective location, project scope and student’s prior experience abroad [60, 61, 65]. Herbst de Cortina et al. argued that in-person, smaller group formats are the most effective way to deliver predeparture resources and designed their curriculum to minimize didactic delivery and optimize interactive small group learning [59].

Length of preparation ranged from 20 min on a web-based training tool [58] to 15 weeks at an in-person course [51]. Fourteen articles did not specify the length of preparation [26, 34–36, 40, 44, 52, 55, 64, 66, 70, 79–81] while eight articles reported that length of preparation was dependent on similar factors mentioned above for delivery modality [41–43, 56, 60, 61, 65, 68].

The majority of studies (*n* = 29) utilized in-person or web-based orientation formats. Five studies found that interactive education tools, such as simulations or computer games, can be effective in preparing students for overseas short-term experiences [50, 55, 58, 64, 67]. One such study evaluated a simulation curriculum designed to elicit the emotional responses similar to what medical residents may face in the field [55]. This study found that simulation-based curriculums are effective because they can be recreated across a wide variety of environments and they allow residents to experience authentic, emotionally-charged scenarios in a controlled environment before traveling abroad to work with patients. Other studies evaluating simulation curriculums found similar benefits and also reported that in addition to increasing knowledge, simulations can improve procedural skill acquisition, problem-solving techniques and increase comfort with diagnosis and management [50, 55, 64, 67]. Butteris et al. recommended that these active preparation modalities be used in addition to traditionally passive forms of preparation. Another study employed an online malaria risk reduction game to increase knowledge and risk perception among students [58]. This study found that a single exposure to a web-based game had significant impact on knowledge in both users with and without previous malaria experience. Hartjes & Baumann suggest that increases in knowledge and awareness of risk attributed to the game have the potential to significantly reduce overall risk of malaria exposure among students traveling abroad. Both Butteris and Hartjes found that interactive education tools can be effective in preparing students for international short-term experiences.

### Other forms of preparation and barriers to conducting preparation

Some articles recognized that pre-departure preparation is not the only mode of preparation for students and that many trainees receive applicable skills, tools, and knowledge through other mechanisms during their education [34, 48, 52]. Medical students reported that working with a diverse group of patients at their home institution prepared them for cross-cultural understanding internationally [41]. It was also noted that many residency programs provide domestic opportunities to learn about issues related to global health through clinical work with diverse patient groups and through exposure to different types of organizations [56]. A number of authors advocated for the integration of global health preparation competencies into other courses and aspects of curricula [52, 66, 69, 84].

Resource constraints for conducting formal pre-departure preparation was a concern for some institutions. Barriers to conducting pre-departure preparation included limited funding [33, 68], lack of buy-in from programs, faculty, and administration [53, 65, 68], and scheduling difficulties [26, 65, 67, 68]. Some pre-departure curricula were started and run by the trainees themselves [53, 72].

## Discussion

This review supports a growing interest in global health training and efforts to prepare trainees for international experiences; the majority (*n* = 33; 60%) of included articles were published recently, between 2014 and 2018. This trend is in-line with the booming growth of global health programs offered across North America. The results of our literature review likely represent the transition of many programs to formal pre-departure curricula and procedures. While it is reassuring that many programs provide pre-travel preparation this systematic review revealed that few institutions have robust guidelines for pre-departure preparation and the content offered to different trainee populations varies widely.

Current curriculum models tend to focus specifically on upcoming short-term experience needs including safety and project-specific knowledge. Far fewer programs addressed thematic domains for longer-term engagement in global health such as mentorship, professionalism, and language. A few articles recognized that knowledge, attitudes, and skills useful in international settings can also be gained through previous experience or through other modes of learning encountered in various training programs. Whether through pre-departure trainings specifically or through other curricula, key thematic domains relevant to learning and partnering in LMIC settings should be addressed and assessed. Given the criticisms global health training programs face such as exploiting poverty and the realities of international partnerships including limited resources for capacity building [7–13], pre-departure training emphasis on the skills and knowledge needed for developing collaboration could help overcome these hurdles in the future. This approach to pre-departure training not only provides trainees with specific skills but also opens the door to longer-term global engagement and helps build a platform for a robust career in global health in the future.

Evaluating programs is crucial for a number of reasons, including program improvement and quantifying program impacts [85]. Only half of the programs that conducted an evaluation of the international elective evaluated and reported on the outcomes and impact of the pre-departure component. These evaluations focused on concepts like trainee knowledge, preparation, and confidence, but most either did not assess or did not report on longer-term outcomes of pre-departure training, such as decision making in unfamiliar situations, increased awareness about available resources, or facilitating relationship building and opportunities. These activities would be considered “deeper learning activities”, which promote critical thinking, communication, and collaboration, by the Hewlett Foundation [86]. The authors argue that these skills are crucial to effective education in the twenty-first century and may be challenging but necessary to assess. To better understand the role of pre-departure training in successful electives, evaluation tools must be rigorous, assessing links between pre-departure training components and outcomes during short-term experiences. For example, evaluation tools could ask students to reflect on whether specific aspects of the pre-departure curriculum such as a module on ethics helped to inform decision-making during a complex situation and if so, how. Data from such evaluations would help institutions tailor pre-departure curricula and add to the body of pre-departure knowledge.

A series of recommendations emerged from evaluation data presented in this review. Programs that offer short-term international experiences should engage with their international partners in the development of both the predeparture material and the content of the experience. This would help better prepare trainees for unique cultural considerations that are specific to the site itself and not just the country or region more broadly. Early and frequent collaboration also demonstrates to trainees the importance of building and maintaining relationships, a vital element of partnership in global health [87]. Critical reflection is also an important component of pre-departure preparation that can be incorporated before, during, and after travel. Reflection is recognized as a necessary attribute for successful health professionals [88–90] and guidelines on professional reflection are being jointly developed by the General Medical Council, the Academy of Medical Royal Colleges, the Conference of Postgraduate Medical Deans, and the Medical Schools Council [91]. A systematic review on reflection among health professionals and trainees found that the anticipation of challenging situations (e.g. research or practice in an unfamiliar setting) can stimulate reflection and that reflection can also be assessed among trainees [92]. Reflective exercises allow trainees to assess their goals and biases and enumerate emerging ethical or cultural challenges. They can also help alleviate reverse culture-shock upon return to the home country. These recommendations can be utilized to develop best-practices in pre-departure preparation, tailored to specific trainee populations.

The modern learner has access to interactive and participatory interfaces which can be tailored to individual needs and provide opportunities for group learning [93]. The advantages of more immersive approaches can include enhancement of conceptual thinking and the provision of simulations of real-life contexts for training [94], both of which are valuable for trainees preparing to travel. Articles in the review also recommended using small-group sessions or case studies to provide space for problem solving, discussion, and feedback. Few pre-departure trainings utilize these tools to engage their learners and more institutions should consider incorporating problem-solving activities, whether case-based or simulation training, into pre-departure curriculums. Problem-solving skills addressed by these approaches can address a variety of thematic domains from project-knowledge to culture shock and professionalism.

## Limitations

The language used to describe traveling to provide medical care is not very specific and overlaps with topics such as medical tourism and travel medicine. We tested different search strategies to attempt to exclude these irrelevant topics but found that we could not do that and still have a comprehensive search strategy. For example, since most of the field programs take place in low and middle-income countries, we tried adding the Cochrane LMIC filter to our search. However, we found that many of the training programs do not specify which countries they are sending their students to, so using the LMIC filter would exclude relevant literature. We did not employ the LMIC filter and instead excluded literature that did not address this inclusion criteria during the screening processes.

## Conclusion

As institutions try to keep pace with growing demand for global health training opportunities they must work with their international partners to develop interactive pre-departure curricula to prepare their trainees for such experiences. Pre-departure training should complement existing institutional approaches to preparing trainees and should be built with both the specific trainee population and international site in mind. Rigorous evaluation of the pre-departure component of short-term experiences is needed to better understand what best prepares trainees to engage in global health.

## Additional files

Additional file 1: Search Terms by Database. This file contains the search terms used in five different databases for the systematic review. (DOCX 13 kb)

Additional file 2: **Table S1.** Articles Identified in the Systematic Review and Key Data. This table includes the articles included in the full-text extraction phase of the systematic review, the authors, year of publication, journal, key competencies addressed, length of preparation, and delivery modality. (DOCX 60 kb)

## Abbreviations

CORD: Council of Emergency Medicine Residency Directors
HEARTT: Health Education and Relief Through Teaching
LMICs: Low-and-Middle-Income Countries
MeSH: Medical Subject Headings
PRISMA: Preferred Reporting Items for Systematic Reviews and Meta-Analyses
