# Supplementary material for: Are you ready? A systematic review of pre-departure resources for global health electives
Source: BMC Med Educ. 2019 May 22;19:166. doi: 10.1186/s12909-019-1586-y (PMC6532266; doi:10.1186/s12909-019-1586-y)
Supplement: Supplementary file 1 — Search Terms by Database. This file contains the search terms used in five different databases for the systematic review. (DOCX 13 kb) [file 12909_2019_1586_MOESM1_ESM.docx]

**Additional file 1: Search Terms by Database**

**Pubmed:**

("Global Health"[mh] OR "global health" OR "international health" OR "abroad" OR "international clinical" OR "medical outreach")

AND ("Education, Medical"[mh] OR "health education"[mh] OR "education" OR "preparation" OR "resources" OR "resource" OR "orientation" OR "training" OR "curriculum" OR "curricula" OR "teaching" OR "teach" OR "Program evaluation"[mh] OR "Students, Health Occupations"[mh] OR "student" OR "students" OR "trainee" OR "trainees" OR "international educational exchange"[mh] OR "international educational exchange")

AND ("Travel"[mh] OR "travel" OR "traveler" OR "travelers" OR "traveling" OR "Field experience" OR "field placement" OR "field program" OR "field programme" OR "field programs" OR "field programmes" OR "pre-departure" OR "predeparture" OR "trip" OR "trips" OR "international program" OR "international programme" OR "international programs" OR "international programmes" OR "international internship" OR "International internships" OR "field practicum" OR "field practica" OR "field practicums")

Searched 2/28/2018, 1062 results

**Embase:**

('global health'/exp OR 'global health' OR 'international health' OR 'abroad' OR 'international clinical' OR 'medical outreach') AND

('medical education'/exp OR 'health education'/exp OR 'education' OR 'preparation' OR 'resources' OR 'resource' OR 'orientation' OR 'training' OR 'curriculum' OR 'curricula' OR 'teaching' OR 'teach' OR 'program evaluation'/exp OR 'health students'/exp OR 'student' OR 'students' OR 'trainee' OR 'trainees' OR 'international educational exchange') AND

('travel'/exp OR 'travel' OR 'traveler' OR 'travelers' OR 'traveling' OR 'Field experience' OR 'field placement' OR 'field program' OR 'field programme' OR 'field programs' OR 'field programmes' OR 'pre-departure' OR 'predeparture' OR 'trip' OR 'trips' OR 'international program' OR 'international programme' OR 'international programs' OR 'international programmes' OR 'international internship' OR 'International internships' OR 'field practicum' OR 'field practica' OR 'field practicums')

Searched 2/28/2018, 1043 results

**Web of Science:**

TS=("global health" OR "international health" OR "abroad" OR "international clinical" OR "medical outreach") AND

TS=("education" OR "preparation" OR "resources" OR "resource" OR "orientation" OR "training" OR "curriculum" OR "curricula" OR "teaching" OR "teach" OR "student" OR "students" OR "trainee" OR "trainees" OR "international educational exchange") AND

TS=("travel" OR "traveler" OR "travelers" OR "traveling" OR "Field experience" OR "field placement" OR "field program" OR "field programme" OR "field programs" OR "field programmes" OR "pre-departure" OR "predeparture" OR "trip" OR "trips" OR "international program" OR "international programme" OR "international programs" OR "international programmes" OR "international internship" OR "International internships" OR "field practicum" OR "field practica" OR "field practicums")

Searched 2/28/2018, 835 results

**Scopus:**

TITLE-ABS-KEY({global health} OR {international health} OR {abroad} OR {international clinical} OR {medical outreach}) AND

TITLE-ABS-KEY({education} OR {preparation} OR {resources} OR {resource} OR {orientation} OR {training} OR {curriculum} OR {curricula} OR {teaching} OR {teach} OR {student} OR {students} OR {trainee} OR {trainees} OR {international educational exchange}) AND

TITLE-ABS-KEY({travel} OR {traveler} OR {travelers} OR {traveling} OR {Field experience} OR {field placement} OR {field program} OR {field programme} OR {field programs} OR {field programmes} OR {pre-departure} OR {predeparture} OR {trip} OR {trips} OR {international program} OR {international programme} OR {international programs} OR {international programmes} OR {international internship} OR {International internships} OR {field practicum} OR {field practica} OR {field practicums})

Searched 2/28/2018, 1443 results

**Ovid Global Health:**

("global health" OR "international health" OR "abroad" OR "international clinical" OR "medical outreach") AND ("education" OR "preparation" OR "resources" OR "resource" OR "orientation" OR "training" OR "curriculum" OR "curricula" OR "teaching" OR "teach" OR "student" OR "students" OR "trainee" OR "trainees" OR "international educational exchange") AND ("travel" OR "traveler" OR "travelers" OR "traveling" OR "Field experience" OR "field placement" OR "field program" OR "field programme" OR "field programs" OR "field programmes" OR "pre-departure" OR "predeparture" OR "trip" OR "trips" OR "international program" OR "international programme" OR "international programs" OR "international programmes" OR "international internship" OR "International internships" OR "field practicum" OR "field practica" OR "field practicums")

Searched 2/28/2018, 239 results
