# Supplementary material for: Are you ready? A systematic review of pre-departure resources for global health electives
Source: BMC Med Educ. 2019 May 22;19:166. doi: 10.1186/s12909-019-1586-y (PMC6532266; doi:10.1186/s12909-019-1586-y)
Supplement: Supplementary file 2 — Table S1. Articles Identified in the Systematic Review and Key Data. This table includes the articles included in the full-text extraction phase of the systematic review, the authors, year of publication, journal, key competencies addressed, length of preparation, and delivery modality. (DOCX 60 kb) [file 12909_2019_1586_MOESM2_ESM.docx]

Additional file 2: **Table S1** Articles identified in the systematic review and key data

| **Author** | **Year** | **Journal** | **Competencies** | **Length of preparation** | **Delivery modality** |
| --- | --- | --- | --- | --- | --- |
| Ahluwalia, P.[17] | 2014 | BioMed Central | Culture  Ethics  Project-specific Knowledge | N/A | Didactic |
| Anderson, K.C. [38] | 2012 | Academic Medicine | Culture  Ethics  Language  Personal Health  Safety | Varies by site | Didactic  Web-based |
| Angelin, M. [39] | 2015 | Medical Education | Emotional Wellness and Culture Shock  Personal Health  Project-specific Knowledge  Safety | N/A | Text |
| Arora, G. [18] | 2017 | Academic Medicine | Personal Health | N/A | Text |
| Barton, E. [55] | 2016 | Western Journal of Emergency Medicine | Culture  Mentorship  Personal Health  Project-specific Knowledge  Safety | N/A | Didactic |
| Bessette, J. [64] | 2017 | Physiotherapy Canada | Culture  Ethics  Personal Health  Project-specific Knowledge  Safety | N/A | N/A |
| Bruno, D.M. [19] | 2015 | Journal of Community Health | Culture  Language  Personal Health  Project-specific Knowledge  Safety | Unspecified | In-person Training |
| Butteris, S.M. [40] | 2014 | Academic Pediatrics | Emotional Wellness and Culture Shock | Unspecified | Simulation |
| Chuang, C. [20] | 2015 | BMC Medical Education | Culture  Language  Personal Health  Safety | Unspecified | Unspecified |
| Currier, C. [21] | 2000 | Journal of Professional Nursing | Culture  Emotional Wellness and Culture Shock  Language  Project-specific Knowledge  Safety | Unspecified | In-person Training |
| Dell, E.M. [22] | 2014 | Internal Journal of Medical Education | Ethics  Safety | N/A | N/A |
| Drain, P.K. [65] | 2009 | Academic Medicine | Culture  Mentorship  Professionalism | Unspecified | In-person Training |
| Dunlap, A. [23] | 2017 | Social Work Education | Culture  Emotional Wellness and Culture Shock  Personal Health  Safety | Weekly (unclear how long) | In-person Training |
| Edwardson, J. [41] | 2015 | International Urogynecology Journal | Culture  Emotional Wellness and Culture Shock  Ethics  Personal Health  Professionalism  Safety | N/A | Text |
| Elit, L. [24] | 2011 | Medical Education | Ethics | N/A | N/A |
| Eneriz-Wiemer, M. [42] | 2012 | Academic Pediatrics | Culture | Varies by training requirement | Didactic  Web-based |
| Evert, J. [66] | 2007 | Family Medicine | Culture  Ethics  Personal Health  Project-specific Knowledge  Mentorship | Unspecified | Didactic |
| Gupta, A. [25] | 2017 | Ochsner Journal | Culture  Language  Personal Health  Project-specific Knowledge | Unspecified | In-person Training  Text |
| Hampton, B.S. [67] | 2014 | American Journal of Obstetrics and Gynecology | Culture  Ethics  Safety | Unspecified | Unspecified |
| Hansoti, B. [68] | 2013 | Academic Emergency Medicine | Culture  Ethics  Mentorship  Professionalism  Safety | Unspecified | In-person Training |
| Haq, C. [43] | 2000 | Family Medicine | Culture  Ethics  Project-specific Knowledge  Safety | 2 weeks | In-person Training |
| Hartjes, L.B. [44] | 2012 | Journal of American College Health | Personal Health  Project-specific Knowledge | 20 minutes | Web-based |
| Herbst de Cortina, S. [45] | 2016 | American Journal of Tropical Medicine and Hygiene | Ethics  Mentorship  Personal Health  Professionalism  Project-specific Knowledge  Safety | 6 hours | In-person Training |
| Imperato, P.J. [26] | 1996 | Journal of Community Health | Culture  Personal Health  Project-specific Knowledge  Safety | Varies by site | In-person Training Text |
| Imperato, P.J. [27] | 2004 | Journal of Community Health | Culture  Personal Health  Project-specific Knowledge  Safety | Varies by site | In-person Training  Text |
| Imperato, P.J. [28] | 2016 | Journal of Community Health | Culture  Personal Health  Project-specific Knowledge  Safety | Varies by site and student previous experience | In-person Training |
| Johnston, N. [69] | 2018 | Journal of Travel Medicine | Personal Health  Safety | N/A | N/A |
| Kalbarczyk, A. [29] | 2015 | Annals of Global Health | Culture  Emotional Wellness and Culture Shock  Mentorship  Professionalism  Project-specific Knowledge  Safety | Unspecified | Unspecified |
| Kittle, N. [30] | 2015 | Journal of Bioethical Inquiry | Culture  Ethics  Project-specific Knowledge | 5 1 hour sessions | Didactic |
| Kumwenda, B. [46] | 2014 | Medical Education | Ethics | Varies by program | Varies by Program/Site |
| Kung, T.H. [56] | 2018 | Medical Education | Culture  Ethics  Project-specific Knowledge  Safety | Unspecified | Unspecified |
| Latta, S. [47] | 2011 | Journal of Midwifery & Women's Health | Ethics  Personal Health  Project-specific Knowledge  Safety | Varies by program | Varies by Program/Site |
| Laven, G. [31] | 2011 | Rural and Remote Health | Culture  Personal Health  Project-specific Knowledge | 40 hours over 3 weeks | Didactic |
| Leow, J.J. [57] | 2012 | Surgeons OverSeas, New York, NY | Personal Health  Language  Mentorship  Project-specific Knowledge | N/A | Text |
| Margolick, J. [48] | 2015 | Canadian Medical Education Journal | Ethics  Project-specific Knowledge | N/A | N/A |
| McCall, D. [58] | 2014 | HealthCare Ethics Committee Forum | Culture  Ethics  Language | N/A | Text |
| Moran, D. [32] | 2015 | Medical Education Online | Ethics | N/A | N/A |
| Peluso M.J. [49] | 2018 | Medical Teacher | Culture  Emotional Wellness and Culture Shock  Ethics  Language  Safety | 90 minutes | In-person Training |
| Perry, E. [33] | 2013 | American Journal of Pharmaceutical Education | Culture  Personal Health  Project-specific Knowledge | 10 weeks | In-person Training |
| Petrosoniak, A. [34] | 2010 | Medical Education | Ethics | N/A | Unspecified |
| Pitt, M.B. [50] | 2016 | Pediatrics | Culture  Project-specific Knowledge | Unspecified | Simulation |
| Purkey, E. [51] | 2016 | BMC Medical Education | Culture  Ethics  Language  Personal Health  Project-specific Knowledge  Safety | Varies by program | Varies by Program/Site |
| Rana, G.K. [52] | 2014 | Journal of the Medical Library Association | Culture  Safety | Unspecified | Didactic  In-person Training |
| Reid, M.J. [35] | 2014 | American Journal of Infection Control | Project-specific Knowledge | 4 hours | Didactic  Simulation |
| Schellhase, E.M. [36] | 2013 | American Journal of Pharmaceutical Education | Culture | 15 weeks | Didactic |
| Schwartz, K.R. [53] | 2015 | The Western Journal of Emergency Medicine | Project-specific Knowledge | 2 days | Didactic  Simulation |
| Seymour, B. [37] | 2013 | Journal of Dental Education | Culture  Ethics  Project-specific Knowledge | Unspecified | In-person Training |
| Sivakumaran L. [54] | 2016 | Canadian Medical Education Journal | Culture  Ethics  Language  Personal Health  Professionalism  Safety | Varies by program | Unspecified |
| Suchdev, P. [59] | 2007 | Ambulatory Pediatrics | Culture  Ethics  Language | N/A | Text |
| Thomas, S.T. [60] | 2018 | Medical Teacher | Culture  Ethics  Language  Mentorship  Personal Health  Project-specific Knowledge  Safety | N/A | N/A |
| Tupesis, J.P. [70] | 2012 | International Journal of Emergency Medicine | Culture  Mentorship  Personal Health  Professionalism  Safety | N/A | N/A |
| VanRooyen, M.J. [61] | 1997 | The Journal of Emergency Medicine | Ethics | N/A | N/A |
| Wallace, L.J. [71] | 2014 | Education for Health | Ethics | N/A | N/A |
| Wiskin, C. [62] | 2018 | Medical Education | Culture  Ethics  Language  Mentorship  Personal Health  Professionalism  Project-specific Knowledge  Safety | Introduce 18 months before departure | Text |
| Wright, D.J. [63] | 2010 | The Journal of Nursing Education | Culture  Language  Mentorship  Personal Health Knowledge  Project-specific  Safety | N/A | In-person Training |
